# A subserosal uterine leiomyoma complicated with intra-abdominal haemorrhage: A case report

**DOI:** 10.1016/j.crwh.2023.e00549

**Published:** 2023-09-23

**Authors:** Shivon Hosein, Sarah Elias, Lorinda Boodram, Vishal Bahall, Lance De Barry

**Affiliations:** Department of Obstetrics and Gynaecology, San Fernando General Hospital, South-West Regional Health Authority, Trinidad and Tobago

**Keywords:** Haemoperitoneum, Uterine leiomyoma, Fibroid, Hysterectomy, Gynaecological surgery

## Abstract

Uterine leiomyomas, or fibroids, are the most common benign tumours of the female genital tract. Although uterine fibroids are commonly associated with menorrhagia, dysmenorrhea, symptomatic anaemia, urinary or bowel symptoms and infertility, intra-abdominal haemorrhage is an exceedingly rare complication. Often, the diagnosis is poorly recognizable based on the patient's clinical presentation and alternative diagnoses such as ruptured ectopic pregnancy, ruptured ovarian cyst or perforated viscus are frequently considered.

Herein, we describe a case of a 50-year-old perimenopausal woman who presented with acute, lower abdominal pain, evolving anaemia, hypovolaemic shock and haemoperitoneum with no discernable source. Emergency exploratory laparotomy confirmed the source of massive haemoperitoneum arising from a ruptured blood vessel supplying a large subserosal uterine leiomyoma and the patient subsequently underwent total abdominal hysterectomy and bilateral salpingo-oophorectomy.

Given the paucity of publications on this clinical entity, the aim of this report is to highlight a rare complication of uterine leiomyomas, its pathophysiological spectrum and its relevance to emergency physicians, general surgeons and gynaecologists.

## Highlights

• Uterine leiomyomas are present in up to 80% of women of reproductive age.
• Haemoperitoneum is an exceedingly rare complication of uterine leiomyomas.
• The diagnosis of haemoperitoneum secondary to uterine leiomyoma is difficult and has to be confirmed intraoperatively.
• Treatment involves a multidisciplinary approach involving emergency doctors, general surgeons and gynaecologists.
• Prompt resuscitation with exploratory surgery involving myomectomy or hysterectomy is the mainstay of treatment.

## 1 Introduction

Uterine leiomyomas are benign tumours of the female genital tract arising from the clonal proliferation of a single myometrial cell [1]. They are estrogen-dependent tumours, bordered by an avascular pseudo-capsule and derive their blood supply from an abnormal plexus of vessels [1]. Leiomyomas commonly affect nulliparous women of African descent and are clinically apparent in up to 80% of women of reproductive age [2]. The size and location of leiomyomas within the uterus are the main determinants of symptomatology and clinical manifestations [2].

Common complications of uterine leiomyomas include menorrhagia, dysmenorrhea, urinary or bowel symptoms, symptomatic anaemia and infertility [3]. Acute complications of leiomyomas are rare and may include torsion of a pedunculated leiomyoma, degeneration and intra-abdominal haemorrhage [4]. Intra-abdominal haemorrhage is an exceedingly rare manifestation of uterine leiomyomas with only a few cases reported in the medical literature since 1902 [5]. Given that haemorrhage associated with leiomyomas mostly occurs per vaginam, alternative diagnoses such as ectopic pregnancy, ruptured ovarian cyst, perforated peptic ulcer or ruptured spleen are often considered, mostly in women of reproductive age [4]. Patients present with sudden onset of severe abdominal pain and profound hypovolaemic shock that necessitates emergency surgical intervention [5]. The diagnosis is often missed preoperatively despite advancements in medical imaging techniques.

Herein we report a case of a perimenopausal woman who presented to the emergency department with an acute abdomen, in hypovolaemic shock and anaemia. Upon laparotomy, intra-abdominal haemorrhage due to subserosal uterine leiomyoma was diagnosed and resolved with total hysterectomy and bilateral salpingo-oophorectomy. This case highlights a rare presentation of a common benign gynaecological tumour and the preoperative dilemmas associated with its diagnosis.

## 2 Case Presentation

A 50-year-old perimenopausal woman, para 1, presented to the emergency department at a tertiary health facility with sudden onset of severe abdominal pain for one day. The pain was described as generalized, with a severity of 9/10 and worsened with movement. The patient sought consultation with her primary health care facility before admission and was transferred considering falling haemoglobin concentration (8.4 g/dL to 7.2 g/dL), with tachycardia and normal blood pressure at that time. The patient denied experiencing vaginal bleeding, nausea, vomiting, abdominal trauma and urinary or bowel symptoms. She had no previous medical, gynaecological or surgical history.

**Fig. 1:**
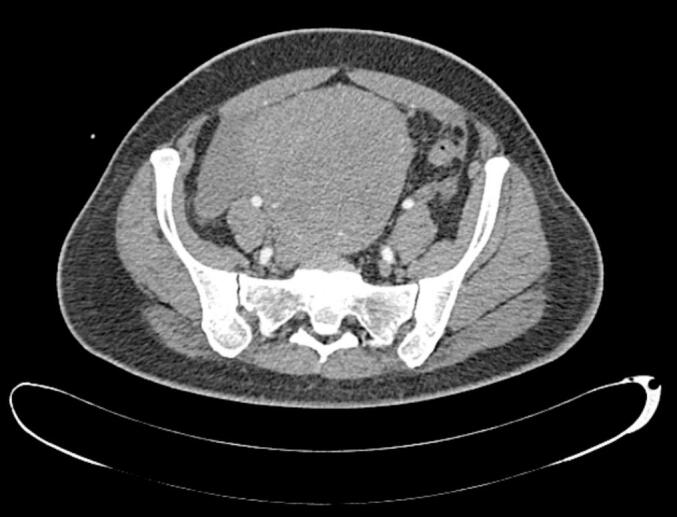
Computed tomography (CT) image of uterus demonstrated fibroid uterus with sinister uterine mass and haemoperitoneum.

On clinical examination, the abdomen was tender in all quadrants, associated with guarding, rigidity and rebound tenderness. Vaginal and speculum examinations were unremarkable and a pregnancy test in the emergency department was negative. Bedside ultrasonography revealed echogenic free fluid in the lower abdomen suggestive of intraperitoneal free fluid. Laboratory investigations highlighted anaemia (haemoglobin 7.2 g/dL, mean corpuscular volume 85 fL, white blood cells 11.78) and normal amylase, lipase and renal and liver function tests. An urgent computed tomography (CT) scan of the abdomen and pelvis was requested to establish the cause of the haemoperitoneum. CT demonstrated moderate haemoperitoneum with no definitive cause, no obvious features to suggest a perforated viscus and a uterine size of 13.6 cm × 9.3 cm × 7.6 cm. The uterus demonstrated mixed densities with some cystic components and differentials included a fibroid uterus versus a sinister uterine mass (Fig. 1).

The general surgeons and gynaecologists were consulted, and the patient was counselled on the CT findings, including the suspicious uterine mass. The patient became progressively tachycardic (pulse 126 beats/min) and hypotensive (blood pressure 95/59 mmHg). Considering the patient's deteriorating status, she was booked by the general surgeons for emergency exploratory laparotomy. The option of a hysterectomy was discussed with the patient in the event that her clinical picture was related to the suspicious uterine mass identified on pelvic CT.

**Fig. 2:**
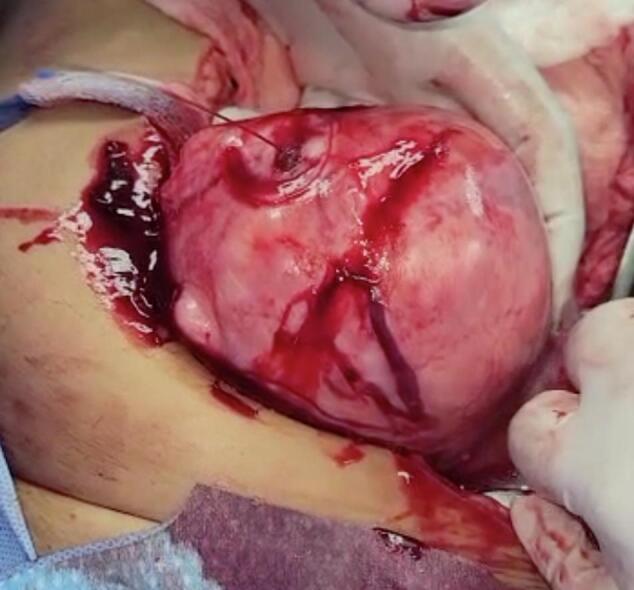
Ruptured blood vessel overlying subserosal leiomyoma.

Under general anaesthesia, the abdomen was opened in layers by an infraumbilical midline incision. Upon abdominal entry, a massive haemoperitoneum was encountered and approximately 1200 ml of blood was evacuated from the abdominal cavity. The intra-abdominal viscera were closely inspected, and normal appearances of the solid and hollow organs were noted. On closer examination of the pelvis, a ruptured tortuous, superficial, dilated artery feeding a 10 cm × 10 cm subserosal anterofundal uterine leiomyoma was noted, with active bleeding (Fig. 2). The pseudo-capsule overlying the leiomyoma was eroded into the feeding blood vessel leading to its rupture and extravasation of blood. The gynaecology team was consulted and upon intraoperative assessment, a decision was made to proceed with a total abdominal hysterectomy and bilateral salpingo-oophorectomy. After successful completion, the vaginal vault was closed with a 1-polyglactin 910 suture. All pedicles were thoroughly inspected to ensure haemostasis and an abdominal drain was inserted. The final estimated blood loss was approximately 2100 ml.

Postoperatively, the patient received two units of packed red cells. Her postoperative course was unremarkable. The abdominal drain was removed after the second postoperative day following negligible output and the patient was discharged in satisfactory condition on the fourth postoperative day. Histopathology confirmed the presence of a single 10 cm × 10 cm uterine leiomyoma that underwent hyaline degeneration.

## 3 Discussion

Intra-abdominal haemorrhage arising from a degenerating uterine leiomyoma is an exceedingly rare complication of uterine fibroids as evidenced by the identification of only 125 cases published in the literature since 1902 [5]. While acute complications of uterine leiomyomas are rare, intra-abdominal haemorrhage often leads to a diagnostic dilemma regarding the source of bleeding [4]. Patients who develop this clinical entity present with hypovolaemic shock, generalized abdominal pain and without a clear pre-operative diagnosis prior to emergency surgery [6]. According to Lim et al., the mortality rate of intra-abdominal haemorrhage as a result of uterine leiomyomas is approximately 3.2% [6].

The main vascular network supplying the uterine fibroid arises from the uterine artery and is often distorted, tortuous and enlarged [7]. The fibroid parenchyma is typically hypovascular, and small centripetal vessels often supply the internal bulk of the fibroid compared to the arteries supplying the peripheral aspect of the fibroid which are usually larger than those supplying the normal myometrium [7]. Moreover, a significant portion of the uterine arterial supply is shunted towards uterine leiomyoma with venous drainage through dilated veins that cross the surface of the fibroid and enter the larger channels at the periphery of the myometrium [8]. These vascular abnormalities produce prominent and weak superficial blood vessels and are particularly associated with fibroids greater than 10 cm in diameter [7].

Although the specific cause of the rupture of superficial vessels supplying uterine fibroids remains unelucidated, several theories have been suggested to explain this rare occurrence. These include bleeding from a subserosal artery, rupture of a subserosal vein draining a uterine fibroid, an avulsed pedunculated fibroid or a ruptured or lacerated uterine fibroid [9]. In most cases, intra-abdominal haemorrhage arises from trauma or torsion of a uterine fibroid [9]. Approximately 76.3% of cases are due to venous bleeds whilst 11.3% are arterial [6]. In the case described, the source of bleeding was arterial in origin. In some cases, no precise aggravating factors have been identified although causes related to overdistension of the superficial vessels leading to rupture have been proposed. These include venous congestion from menstruation, pregnancy, defecation, violent coitus or contact sports [6].

In most cases, a clear pre-operative diagnosis or source of bleeding is often unknown [10]. Patients typically initially present with abdominal pain: 83% with sudden onset of generalized abdominal pain and 17% with a pain that worsens over the course of 3–5 days [6]. Hypovolemic shock is commonly associated with intra-abdominal haemorrhage arising from uterine leiomyomas [6]. Cases of cardiovascular collapse are rare but have been documented.

Blood investigations usually demonstrate an evolving anaemia whilst point-of-care ultrasonography often highlights abdominopelvic-free fluid and can identify the presence of uterine fibroids [11]. Computed tomography (CT) imaging of the abdomen and pelvis, with contrast, remains the best diagnostic modality available, although it often does not yield a definitive diagnosis [12]. In such clinical circumstances, the presence of a uterine fibroid in the visage of intra-abdominal haemorrhage of unknown origin may be considered a “red herring” [6]. Differential diagnoses such as ruptured ectopic pregnancy, ovarian torsion, haemorrhagic corpus luteum, ruptured ovarian cyst, splenic rupture or rupture of an aortic aneurysm are differential diagnoses that are often preferentially considered [4].

Prompt resuscitation and exploratory surgery by either laparotomy or laparoscopy is the mainstay of life-threatening intra-abdominal haemorrhage [13]. The definitive management is strictly surgical, in the form of myomectomy or hysterectomy [9]. Myomectomy is preferred in nulliparous women of reproductive age and in those wishing to retain fertility [13]. In most cases, exploratory surgery is initially undertaken by a general surgical team and once an intraoperative diagnosis is confirmed, the gynaecologist is consulted to approach the myomectomy or hysterectomy [9]. Furthermore, access to a blood bank and intensive care monitoring improves the outcomes of patients diagnosed with this unique and life-threatening clinical entity. Given the age of the patient described here, a hysterectomy was performed.

In conclusion, spontaneous or traumatic intra-abdominal haemorrhage in women of reproductive age is commonly associated with ruptured ectopic pregnancy, ruptured corpus luteum or non-gynaecological causes of haemoperitoneum and is rarely attributed to uterine fibroids. Clinicians should demonstrate a high index of suspicion for haemoperitoneum arising from uterine fibroids when a woman of reproductive age presents with acute abdominal pain, hypovolaemic shock and haemoperitoneum of unknown origin. Resuscitative measures, prompt exploratory surgery and a multidisciplinary operative approach among surgeons, gynaecologists and emergency doctors are imperative to improve patient outcomes. Due to the limited publications on this clinical entity, we expect to raise awareness of the rare manifestation of the most common benign tumour of the female genital tract.
